# Newly identified c-di-GMP pathway putative EAL domain gene *STM0343* regulates stress resistance and virulence in* Salmonella* enterica serovar Typhimurium

**DOI:** 10.1186/s13567-024-01437-0

**Published:** 2025-01-15

**Authors:** Kaifeng Chen, Lili Li, Nanwei Wang, Zhouping Zhou, Peng Pan, Chenggang Xu, Dage Sun, Jiayi Li, Changzhi Dai, Dai Kuang, Ming Liao, Jianmin Zhang

**Affiliations:** 1 National and Regional Joint Engineering Laboratory for Medicament of Zoonoses Prevention and Control, Key Laboratory of Zoonoses, Ministry of Agriculture, Key Laboratory of Zoonoses Prevention and Control of Guangdong Province, Key Laboratory of Animal Vaccine Development, Ministry of Agriculture, College of Veterinary Medicine, South China Agricultural University, Guangzhou, 510642 China; 2 Institute of Animal Health, Guangdong Academy of Agricultural Sciences, Guangzhou, 510640 China; 3 National Health Commission (NHC) Key Laboratory of Tropical Disease Control, School of Tropical Medicine, Hainan Medical University, Haikou, China

**Keywords:** *S.* Typhimurium, c-di-GMP, EAL, stress resistance, virulence

## Abstract

*S.* Typhimurium is a significant zoonotic pathogen, and its survival and transmission rely on stress resistance and virulence factors. Therefore, identifying key regulatory elements is crucial for preventing and controlling *S.* Typhimurium. We performed transcriptomic analysis and screened for a c-di-GMP pathway key gene *STM0343*, a putative EAL domain protein with an unknown function. Our findings revealed that the deletion of this gene (269Δ*STM0343*) led to a 29.85% increase in c-di-GMP. In terms of stress resistance, the strain 269Δ*STM0343* showed significant improvements compared to the wild strain WT269. Specifically, it exhibited increases of 95.74% in extracellular protein and 35.96% in exopolysaccharide production by upregulating the expression of relevant genes. As a result, the biofilm formation ability of 269Δ*STM0343* was enhanced by 21.54%, accompanied by a more pronounced red, dry, and rough colony morphology. 269Δ*STM0343* also showed a 19.03% decrease in motility due to the downregulation of *flhD* expression. As a result, 269Δ*STM0343* increased resistance to various antibiotics, as well as to acidic conditions, oxidative stress, and disinfectants. In terms of virulence, compared to WT269, the adhesion and invasive ability of 269Δ*STM0343* to HeLa cells was enhanced by onefold and 25.67%, respectively. In in vivo experiments, mice challenged with 269Δ*STM0343* experienced greater weight loss, and the bacterial loads in the spleen, liver, and intestines were elevated by fivefold, 30-fold, and 21-fold, respectively, accompanied by more severe pathological damage. Mechanistic studies revealed that the adhesion and invasion capacities of 269Δ*STM0343*Δ*CsgB* decreased by 29.41% and 68.58%, respectively, compared to 269Δ*STM0343*. Additionally, LacZ gene reporting indicated that *STM0343* inhibited the expression of *CsgB.* This suggests that *STM0343* suppresses virulence by downregulating *CsgB* expression. This study provides insights into the regulatory mechanisms by which *STM0343* reduces the stress resistance and pathogenicity of *S.* Typhimurium.

**Supplementary Information:**

The online version contains supplementary material available at 10.1186/s13567-024-01437-0.

## Introduction

*Salmonella* is a significant zoonotic pathogen that poses serious risks to both animal and human health [1]. *S.* Typhimurium is one of the most important serotypes within the *Salmonella* genus, known for its high pathogenicity. It typically causes self-limiting illnesses such as vomiting, diarrhoea, and abdominal pain; however, severe cases can result in bacteremia and even death. Globally, *S.* Typhimurium is responsible for an estimated 21.7 million illnesses and 217 000 deaths [2].

Additionally, *S.* Typhimurium exhibits strong resistance to stress, including developing resistance to antibiotics and fungicides [3]. It also adapts to the host’s internal environment to facilitate growth and proliferation [3]. As a result, *S.* Typhimurium has become widespread and is the dominant serotype in many countries and regions, including China [4], Australia [5], and Africa [6]. Its strong adaptability to stress, high morbidity rates, and the challenges associated with prevention and control have established *S.* Typhimurium as a significant zoonotic pathogen concerning public health.

C-di-GMP is a second messenger that regulates various cellular processes in bacteria, contributing to stress adaptation and virulence [7]. It plays a crucial role in biofilm formation and motility in pathogens such as *Escherichia coli* [8], *Vibrio vulnificus* [9], and *P. aeruginosa* [10]. The synthesis and degradation of c-di-GMP are controlled by diguanylate cyclases (DGCs) and phosphodiesterases (PDEs), which have GGDEF and EAL or HD-GYP domains, respectively [11, 12].

Different genes within this pathway have distinct regulatory functions. For example, *AdrA,* which contains a GGDEF domain, can promote biofilm formation by regulating the synthesis of exopolysaccharides [13], while *STM1344,* with an EAL domain, can inhibit the motility of *S.* Typhimurium [14]. The EAL-like protein *STM1697* plays a role in regulating virulence [15]. In *S.* Typhimurium, a total of 22 genes associated with the c-di-GMP pathway have been identified [16].

Therefore, it is essential to explore new c-di-GMP pathway genes and understand their functions to gain insights into the gene regulatory network of *S.* Typhimurium. Our laboratory previously identified 20 differentially expressed genes in the c-di-GMP pathway by analysing strains with varying abilities to form biofilms [17]. Among these genes, *STM0343*, a hypothetical EAL domain gene, has an unclear role in biofilm formation, stress adaptation, and virulence in *S.* Typhimurium. Investigating *STM0343* will enhance our understanding of the c-di-GMP regulatory network and identify potential control targets in *S.* Typhimurium.

Based on this, this study examined the role of the hypothetical EAL domain gene *STM0343* in regulating c-di-GMP levels. Through phenotypic characterisation and mechanistic investigations, the study revealed how *STM0343* affects biofilm formation, stress resistance, and virulence in *S.* Typhimurium. These findings lay the groundwork for screening potential targets and developing strategies for the prevention and control of *S.* Typhimurium.

## Materials and methods

### Strains, primers, and plasmids

The *Salmonella* strains utilised in this study included wild-type *S.* Typhimurium WT269, which was isolated from the joint fluid of patients at a hospital in Shanghai. HeLa cells were employed to evaluate the strains’ adhesion and invasive capabilities. Competent *E.* coli DH5α (Takara, China) was used for cloning experiments. All primers and plasmids used in this study are listed in Additional files 1, 2, and 3.

### Construction of gene deletion mutant strains (269Δ*STM0343*/269Δ*CsgB*/269Δ*CsgB*Δ*STM0343*) and *STM0343* complemented transformant (269Δ*STM0343*R)

The λRed-mediated mutagenesis method [18] used to generate the 269Δ*STM0343*/269Δ*CsgB/269ΔCsgBΔSTM0343* mutant strains. In this process, the kanamycin resistance gene, which is flanked by FRT sites, along with the upstream and downstream genes of *STM0343/CsgB*, was amplified using the primers *STM0343-L1-F/R* and *CsgB-L2-F/R*, respectively. The plasmid pKD4 served as the template for this amplification.

These amplified fragments were then transferred into WT269 competent cells, which had been induced with L-arabinose at a final concentration of 100 mmol/L. This induction allowed for the expression of pKD46, thereby facilitating recombination with the bacterial chromosomes. Plasmid pCP20 was subsequently transformed into homologous recombinant cells to remove the kanamycin cassette. The successfully mutated strains were confirmed using the primers *STM0343-JD-F/R* and *CsgB-JD-F/R* and were cultured at 42 °C to eliminate pCP20. The strain 269Δ*CsgB*Δ*STM0343* was constructed by deleting the *CsgB* gene, using 269Δ*STM0343* as the genetic background. To create the *STM0343* complemented transformant (269Δ*STM0343R*), the *STM0343* expression vector was constructed with pBAD-HisA and then electrotransferred into 269Δ*STM0343*.

### Determination of growth curves

The recovered WT269, 269Δ*STM0343*, and 269Δ*STM0343R* single colonies were picked and inoculated into LB broth, respectively. They were incubated overnight at 37 °C, with shaking at 180 rpm. The following day, the overnight culture was diluted into fresh LB broth, and the optical density at 600 nm (OD_600nm_) was adjusted to 0.1. The culture was continuously incubated at 37 °C with shaking at 180 rpm for 20 h. OD_600nm_ values were measured at one-hour intervals, and the growth curves were plotted based on these measurements [19]. This experiment included three biological replicates.

### Determination of bacterial c-di-GMP content

The extraction of c-di-GMP was performed using the method described by Spangler et al. [20], with minor modifications. First, the overnight cultures of WT269, 269Δ*STM0343*, and 269Δ*STM0343R* were diluted 1:100 into fresh LB broth and incubated for 8 h at 37 °C, with shaking at 180 rpm. The bacterial solution was then centrifuged, and the supernatant was discarded. The precipitate was washed three times with PBS, after which the bacteria were resuspended with PBS, adjusting the OD_600nm_ to a consistent value. The organisms were broken down using a crushing method at 200 W for 10 s, followed by a 10 s pause, for a total processing time of 15 min. Afterwards, the mixture was centrifuged at 13 000 rpm for 2 min. The supernatant was discarded, and the remaining precipitate was washed with PBS. The precipitate was then resuspended in 2 mL of PBS and subjected to a water bath at 100 °C for 5 min. Following this, ice-cold ethanol (final concentration of 65%) was quickly added and extracted for 15 s. The mixture was then centrifuged at 13 000 rpm for 2 min, and the supernatant was collected to obtain the c-di-GMP sample. The c-di-GMP content in the sample was then measured using an ELISA kit from Shanghai Hengyuan Biological Technology Co., Ltd., and the results were quantified based on a standard curve. This experiment was conducted with three biological replicates.

### Crystal violet staining to assess biofilm formation ability

Crystalline violet staining assay [21] was used to quantify the biofilm formation ability of WT269, 269Δ*STM0343*, and 269Δ*STM0343R*. The overnight cultured strains were diluted 1:100 in fresh unsalted LB broth. The diluted cultures were then inoculated into 96-well plates and incubated for 48 h at 30 °C. After discarding the planktonic bacteria, the cells in the wells were washed and fixed. A crystal violet solution was added to each well and allowed to stain for 15 min. The unbound dye was gently removed, and 200 µL of 33% acetic acid was added to each well to dissolve the crystal violet. Finally, the biofilm biomass was estimated by measuring the optical density at 595 nm (OD_595 nm_) using a microplate reader. This experiment was conducted with three biological replicates.

### Observation of colony morphology

According to the previously reported method [22], 10 μL of the overnight cultured strains were inoculated onto pre-prepared salt-free LB agar plates containing 40 mg/mL of Congo red and 20 mg/mL of Coomassie Brilliant Blue G. The agar plates were then incubated at 27 °C for 5 days. At the end of the incubation period, the colours and roughness of the colonies on the agar plates were observed. This experiment was conducted with three biological replicates.

### Quantification of the extracellular matrix

To determine the content of the biofilm-associated extracellular matrix, we followed method described by Dressaire et al. [23].

Colonies cultured on LB agar plates for 18 h were resuspended in 0.9% NaCl solution. After centrifugation, we collected the supernatant and mixed with it 99% cold ethanol. The mixture was then centrifuged at maximum speed for 20 min at 4 °C. The resulting pellet was dried and resuspended with ddH_2_O. Finally, we measured the concentrations of proteins and DNA in the samples using a NanoDrop 1000 (NanoDrop Technologies). To quantify the exopolysaccharide, 100 μL of the sample was taken and diluted to 1 mL with sterile deionised water. Next, a phenol solution was added, followed by concentrated sulfuric acid, with thorough mixing after each addition. The sample was allowed to stand for 10 min and then incubated in a water bath at 65 °C water bath for 18 min. After incubation, it was quickly cooled by placing it in ice until it reached room temperature. Finally, the absorbance at 490nm (OD_490nm_) was measured using a microplate reader, and the concentrations were determined based on a glucose calibration curve. This experiment was conducted with three biological replicates.

### Motility assays

The motility of the strains WT269, 269Δ*STM0343,* and 269Δ*STM0343*R was assessed using the 0.3% agar plate method [21]. A volume of 10 μL from overnight cultures was inoculated in the centre of 0.3% semi-solid LB agar plates. The plates were then incubated at 37 °C, and the motility diameter of the strains in the agar was determined after 6 h. This experiment was conducted with three biological replicates.

### RNA extraction and qRT-PCR (quantitative real-time reverse transcription PCR)

Overnight cultured WT269, 269Δ*STM0343*, and 269Δ*STM0343R* were diluted into fresh LB medium at a 1:100 ratio. The cultures were incubated at 37 °C with agitation at 180 rpm for 4 h. Following incubation, total bacterial RNA was then extracted using the RNA Extraction Kit (Omega Bio-tek, Norcross, GA, USA). The extracted RNA was then reverse transcribed into cDNA using the HiScript® III RT SuperMix for qPCR (+ gDNA WIper) according to the provided instructions. Finally, the expression levels of the genes *BcsA*, *BcsB*, *CsgA*, *CsgB*, which are involved in biofilm formation, as well as the genes *fliA*, *flhC*, *flhD*, which are related to bacterial motility, were analysed. This was done using a ChamQ Universal SYBR qPCR Master Mix kit (Novozan Biotechnology Co., Ltd., Nanjing, China) through qRT-PCR with the primers listed in Additional file 2. The 16S rRNA genes were used as an internal control for normalisation. This experiment was conducted with three biological replicates.

### Determination of antibiotic susceptibility by broth microdilution method

Minimum Inhibitory Concentrations (MICs) of WT269, 269Δ*STM0343*, and 269Δ*STM0343R* against 15 antibiotics were determined using the broth microdilution method recommended by the Clinical and Laboratory Standards Institute (CLSI). For the MIC assay, single colonies were transferred into MH broth and incubated at 37 °C, shaking at 180 rpm for 4 h until the OD_600nm_ reached 0.5. A 1:100 dilution of the bacterial culture was then prepared in fresh MH broth. In a 96-well plate, 20 μL of antibiotics at an initial concentration of 10,240 μg/mL was added to 180 μL of MH broth. The antibiotics were then sequentially diluted in a twofold manner. Finally, 100 μL of the diluted bacterial solution was added to the various concentrations of antibiotics. The MIC values were measured after 16–18 h of static incubation at 37 °C. This experiment was conducted with three biological replicates.

### Acid and oxidative stress assay

Overnight cultures of WT269, 269Δ*STM0343,* and 269Δ*STM0343R* were centrifuged at 6000 rpm for 5 min. The supernatant was discarded, and the organisms were washed three times with sterile PBS. After washing, the cells were resuspended in LB supplemented with acid (final pH = 4.4), oxidative (5 mM H_2_O_2_), and SDS disinfectant (0.25%). The OD_600nm_ of the bacterial suspension was adjusted to 0.1. The cultures were then incubated at 37 °C with agitation at 220 rpm. OD_600nm_ values were measured at 1-h intervals and growth curves were plotted [19]. This experiment was conducted with three biological replicates.

### Cell adhesion and invasion assay

Overnight cultured strains were diluted 1:100 into fresh LB broth and incubated at 37 °C, with agitation at 220 rpm until the OD_600nm_ was 0.6–0.8. After reaching the desired density, the bacterial culture was washed three times with PBS. The bacteria were then resuspended in DMEM. To assess cell adhesion, the resuspended bacteria were co-cultured with HeLa cells for 2 h at a multiplicity of infection (MOI) of 1:100. Subsequently, the cells were washed thrice with PBS to remove non-adherent bacteria. They were then treated with 0.1% Triton X-100 for 15 min to lyse the cells. The resulting cell lysates were serially tenfold multiplicative diluted with PBS, and colonies from the diluted lysates were counted using the plate decantation method. After co-culturing the bacteria with the cells for the cell invasion assay, the cells were washed three times with PBS containing 100 μg/mL gentamicin. Following the washes, DMEM containing 100 μg/mL of gentamicin was added, and the mixture was incubated for 1 h to eliminate any extracellular bacteria. Finally, the colonies were counted using the same method as described above. This experiment was conducted with three biological replicates.

### In vivo infection of C57BL/6 mice

Animal experiments were conducted following the previously described protocols [1]. In brief, four-week-old SPF-grade C57BL/6 mice, purchased from the Guangdong Medical Laboratory Animal Center, Guangzhou, China, were divided into four groups of seven animals each. The mice were fasted for 24 h and water-deprived for 12 h before the challenge. They were then administered 1 × 10^8^ CFU (100 μL) of WT269, 269Δ*STM0343*, 269Δ*STM0343R* by gavage, respectively. The last group of mice were each gavaged with 100 μL of PBS as a control.

The body weight of each mouse was recorded continuously for 7 days after the challenge, and on the seventh day, the mice were euthanised. Their intestines, livers, and spleens were then collected for bacterial colony counting. The bacterial load was calculated as bacterial CFU in 1 g of tissue (CFU/g). Additionally, pathological sections of the intestine, liver, and spleen were prepared for analysis. Based on previously reported methods, we conducted quantitative pathological scoring of the tissue sections. The scoring criteria were as follows: grade 0 for no damage, grade 1 for < 25% damage, grade 2 for 25–49% damage, grade 3 for 50–75% damage, grade 4 for > 75% damage [24]. Additionally, each mouse's body weight measurements and bacterial load counts were carried out in triplicate.

### LacZ reporter gene fusion assay

In the previous report [25], the promoter of *CsgB* was amplified using the primers *CsgB*-pro-F/R. A LacZ gene reporter system targeting *CsgB* was constructed using pRCL as a vector. This constructed vector was then electro-transferred into WT269, 269Δ*STM0343*, and 269Δ*STM0343R.* The strains were incubated at 37 °C with shaking at 180 rpm for 8h. After adjusting the OD_600nm_ to a consistent level, the cultured strains were disrupted using sonication. The OD_600nm_ of the resulting liquid was measured, followed by centrifugation at 4 °C and 15 000 × *g* for 20 min. The supernatant was then collected. Finally, the activity of β-galactosidase in the supernatant was assessed using a kit from BOXBIO (Beijing, China). This experiment was conducted with three biological replicates.

### Statistical analysis

All experiments in this study were performed in triplicate, and the data were analysed and visualised using GraphPad Prism V8.0 (GraphPad Inc., La Jolla, CA, USA). Multiple t-tests were performed to assess differences in the data. In the figures, asterisks denote statistical significance as follows: “*” indicates *P* ≤ 0.05, “* *” indicates *P* ≤ 0.01, and “* * *” indicates *P* ≤ 0.001.

## Results

### *STM0343* decreased the level of c-di-GMP

**Figure 1:**
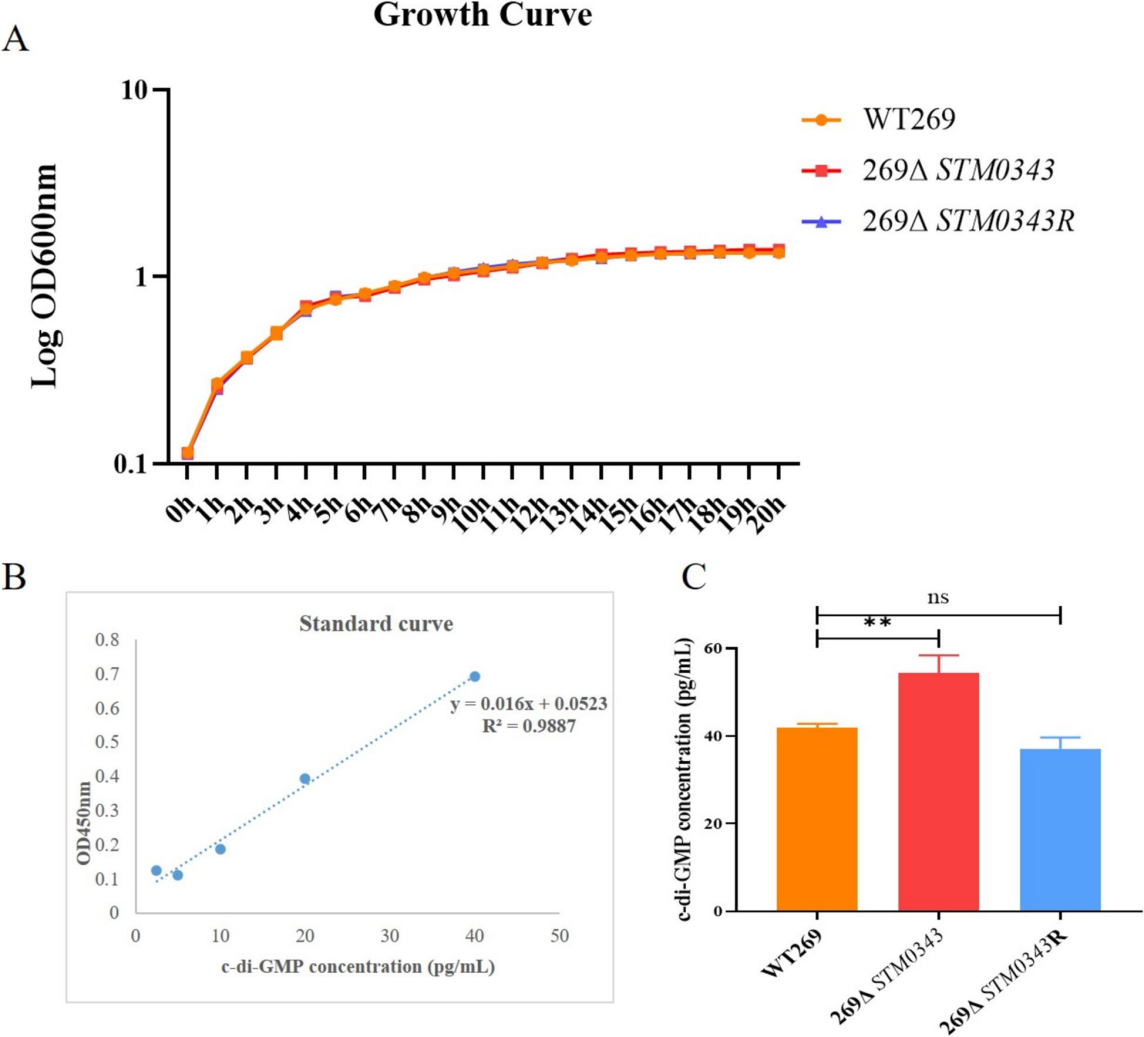
**Detection of c-di-GMP content in bacteria.** **A** Growth curves of WT269, 269∆*STM0343*, 269∆*STM0343*R. Bacteria with an initial OD_600nm_ of 0.1 were cultured in LB broth, and OD_600nm_ was measured hourly for a duration of 20 h. **B** A standard curve depicting c-di-GMP concentration in relation to OD_450nm_ value, using c-di-GMP standards and the method outlined in the ELISA kit. The standard curve equation derived from this data is y = 0.016x + 0.0523 (R^2^ = 0.9887). **C** The c-di-GMP content of WT269, 269Δ*STM0343*, 269Δ*STM0343*R was detected according to the established standard curve, **P < 0.01.

The study conducted a growth curve analysis and found that *STM0343* did not significantly affect the growth of *S.* Typhimurium (Figure 1A). Since *STM0343* is a putative EAL domain protein, the present study investigated its effect on c-di-GMP levels. By measuring c-di-GMP levels in the wild strain WT269, as well as in the constructed deletion strain 269Δ*STM0343* and the complementation strain 269Δ*STM0343R* (Additional file 4), it was discovered that the c-di-GMP content in the *STM0343* deletion strain 269Δ*STM0343* was significantly increased by 29.85% (*P* < 0.01) compared to WT269 (Figures 1B and C). This suggests that *STM0343* likely functions as a phosphodiesterase, degrading intracellular c-di-GMP.

### *STM0343* reduces biofilm formation ability by inhibiting the production of extracellular proteins and exopolysaccharide

**Figure 2:**
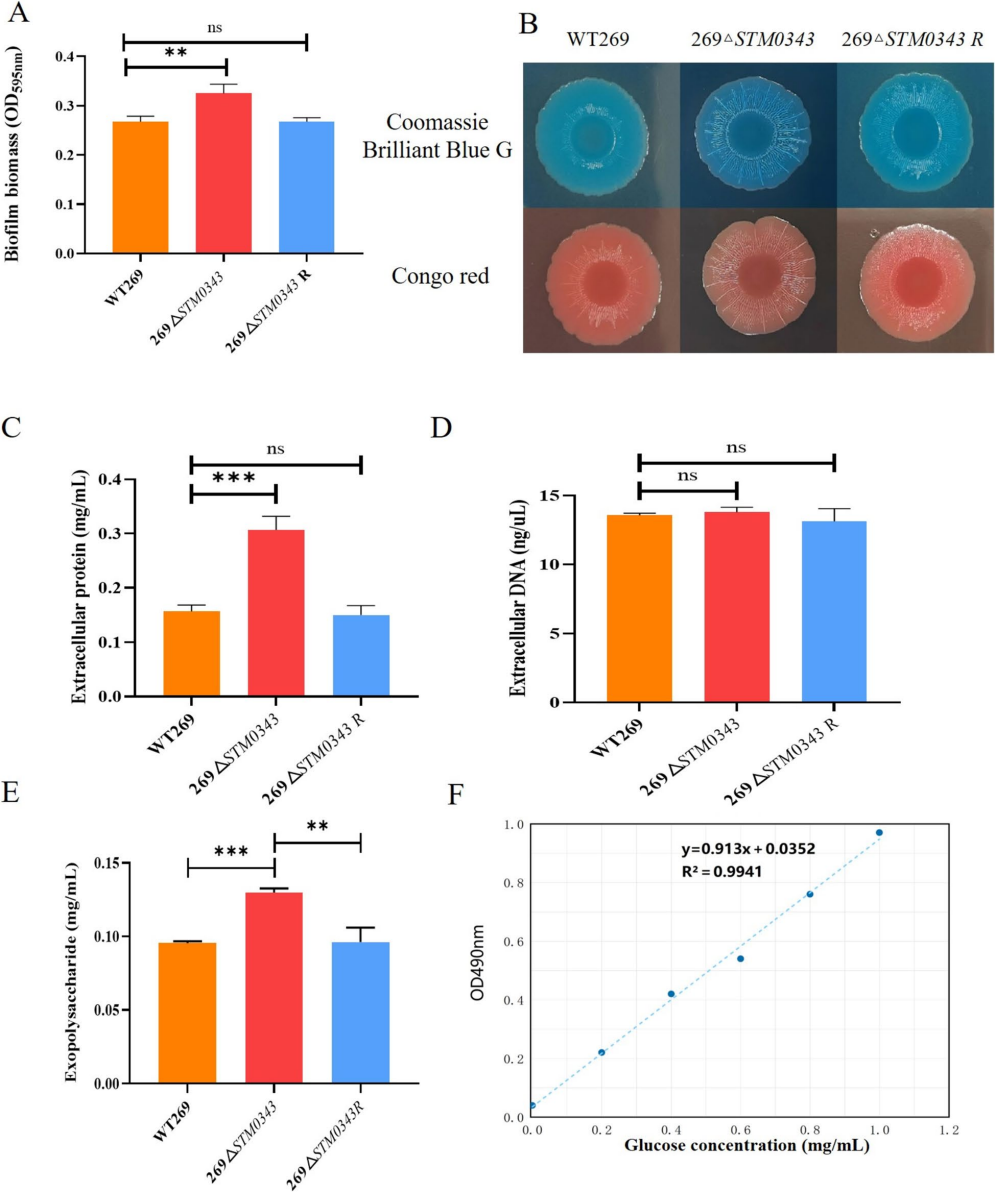
***STM0343* reduces the biofilm formation ability of* S.* Typhimurium.** **A** Detection of biofilm formation ability of the strains by crystal violet staining method; **B** Colony morphology was observed by incubation on Congo red (40 mg/mL) and Coomassie Brilliant Blue G (40 mg/mL) plates; **C**, **D** and **E** are the identification of extracellular protein, extracellular DNA and exopolysaccharide content of the strains, respectively; **F** Standard curve for the determination of exopolysaccharide content, **P < 0. 01, ***P < 0.001.

In this study, we investigated the effect of *STM0343* on biofilm formation ability using crystal violet staining. It was found that the biofilm formation ability of the 269Δ*STM0343* strain was enhanced by 21.54% compared to the wild strain (*P* < 0.01, Figure 2A). This finding was further supported by the observation that the deletion of *STM0343* led to a rougher colony morphology on Coomassie Brilliant Blue and Congo Red plates compared to the wild strain. The 269Δ*STM0343* strain exhibited a rough texture that spread across the surface of the moss and extended to its edges, resulting in wrinkled and irregular edge formations with increased magnitude. In contrast, the wild-type strain and the complemented strain (269Δ*STM0343R*) exhibited smooth characteristics, unlike the roughness observed in the other samples (Figure 2B). These findings indicate that *STM0343* plays a role in inhibiting biofilm formation.

To clarify the mechanism, we analysed the content of biofilm-associated extracellular matrix in WT269, 269Δ*STM0343,* and 269Δ*STM0343R*. Compared to the wild strain, the extracellular protein content in the strain 269Δ*STM0343* was significantly higher, showing an increase of 95.74% (*P* < 0.0001, Figure 2C). Additionally, exopolysaccharide content was determined using the constructed standard curve equation y = 0.913x + 0.0352 (R^2^ = 0.9941). It was found that the exopolysaccharide content in strain 269Δ*STM0343* was elevated by 35.96% compared to WT269 (Figures 2E and F). However, there were no significant differences in the extracellular DNA components between 269Δ*STM0343* and WT269 (*P* > 0.05, Figure 2D). At the gene level, the expression levels of extracellular protein-coding genes (*CsgA* and *CsgB*) and genes related to exopolysaccharide production (*BcsA* and *BcsB*) were significantly higher in 269Δ*STM0343*, as analysed by QRT-PCR (Additional file 5). These findings suggest that *STM0343* primarily reduces biofilm formation by inhibiting the production of extracellular proteins and exopolysaccharides.

### *STM0343* enhanced motility by promoting the expression of flagellar genes

**Figure 3:**
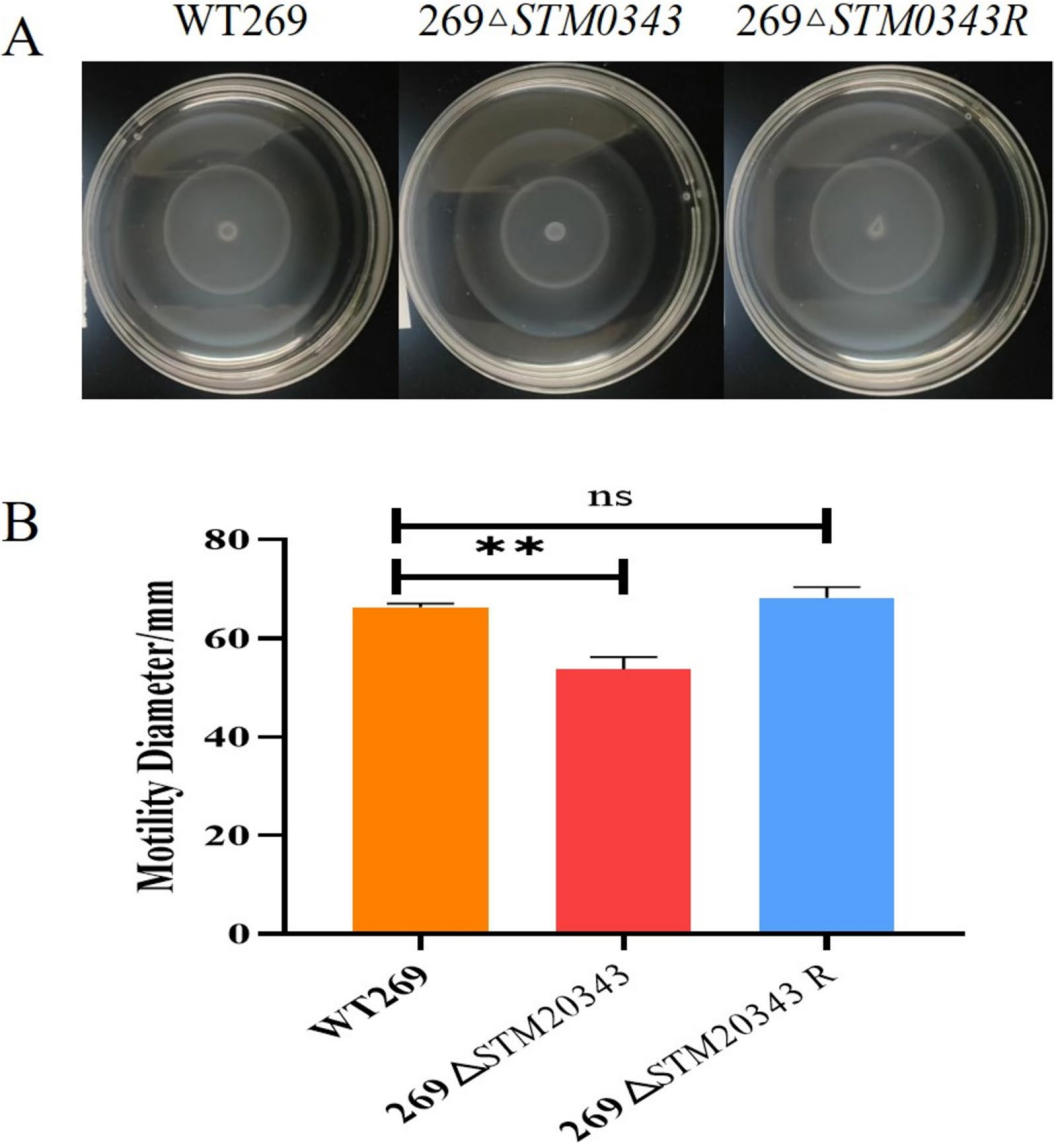
**The effect of *STM0343* on the motility of *S.* Typhimurium.** **A** Motility distribution of WT269, 269Δ*STM0343* and 269Δ*STM0343R* on 0.3% agar plates; **B** Measurement of motility diameter of WT269, 269Δ*STM0343* and 269Δ*STM0343R*, **P < 0.01.

To investigate the effect of *STM0343* on bacterial motility, we measured the motility diameter of bacteria on 0.3% LB agar plates. The results showed that the motility diameter of 269∆*STM0343* was reduced by 19.03% (*P* < 0.01) at the sixth hour compared to the wild strain WT269 (Figure 3). Additionally, at the gene level, the expression of the flagellar synthesis gene *flhD* (Additional file 5), which is associated with motility, was significantly reduced in the absence of *STM0343* compared to the wild strain. This indicates that the deletion of *STM0343* impairs the motility of *S.* Typhimurium by inhibiting flagellar synthesis.

### Expression of *STM0343* reduces the stress tolerance response of *S.* Typhimurium

Given that *STM0343* is a putative c-di-GMP pathway gene and considering its regulatory role in biofilm formation, this study further investigates its function in *S.* Typhimurium’s response to various bactericidal environments.

To begin with, an antibiotic susceptibility test was conducted on 269Δ*STM0343*, WT269, and 269Δ*STM0343R*, involving 15 different antibiotics. The results revealed that 269Δ*STM0343* exhibited a sevenfold reduction in susceptibility to ceftazidime and ceftiofur, along with a onefold reduction in susceptibility to cefepime, cefotaxime, tetracycline, doxycycline, kanamycin, streptomycin, and nalidixic acid, when compared to the wild-type strain WT269 (Table 1).

**Table 1:** **Effect of** ***STM0343*** **on antibiotic susceptibility**

	FEP	CTX	EFT	CAZ	TET	DOX	KAN	GEN	STR	FFC	CHL	NAL	CIP	NOR	OFX
269	4	4	16	8	1	2	2	0.5	8	2	4	8	0.5	0.5	0.5
269Δ*STM0343*	8	8	128	64	2	4	4	0.5	16	2	4	16	0.5	0.5	0.5
269269Δ*STM0343*R	4	4	16	8	1	2	2	0.5	8	2	4	8	0.5	0.5	0.5

FEP, Cefepime; CTX, Cefotaxime; EFT, Ceftiofur; CAZ, Ceftazidime; TET, Tetracycline; DOX, Doxycycline; KAN, Kanamycin; GEN, Gentamycin; STR, Streptomycin; FFC, Florfenicol; CHL, Chloramphenicol; NAL, Nalidixic acid; CIP, Ciprofloxacin; NOR, Norfloxacin; OFX, ofloxacin.

During stress tests involving acid, oxidative, and disinfectants, the growth of the strain was not inhibited after the deletion of *STM0343;* in fact, it was significantly faster*.* Additionally, the mutant strain exhibited higher OD_600nm_ values than the wild-type strain during acid stress starting from the 8th hour, oxidative stress from the 7th hour, and SDS stress from the very first hour. This trend persisted until the 24th hour.

**Figure 4:**
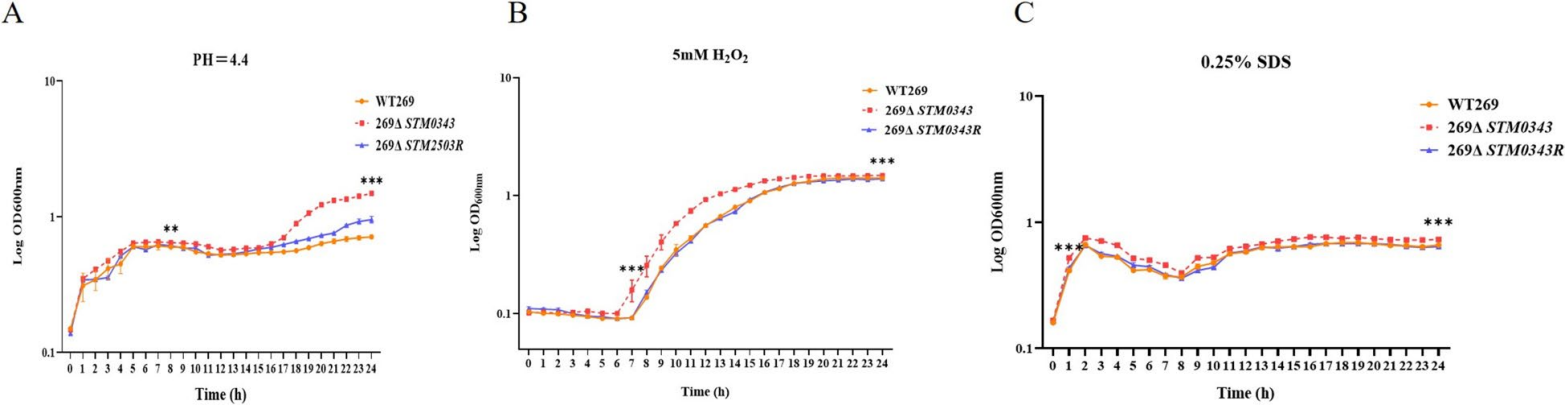
**Role of *STM0343* in the stress response of *S.* Typhimurium.** **A**–**C** are the effects of *STM0343* on acid tolerance, oxygen tolerance, and SDS disinfectant tolerance of *S.* Typhimurium, respectively. *P < 0.05, **P < 0.01, ***P < 0.001.

These findings suggest that *STM0343* reduces the stress resistance of *S.* Typhimurium (Figure 4).

### Deletion of *STM0343* elevates the ability of *S.* Typhimurium to invade and adhere to HeLa cells in vitro

**Figure 5:**
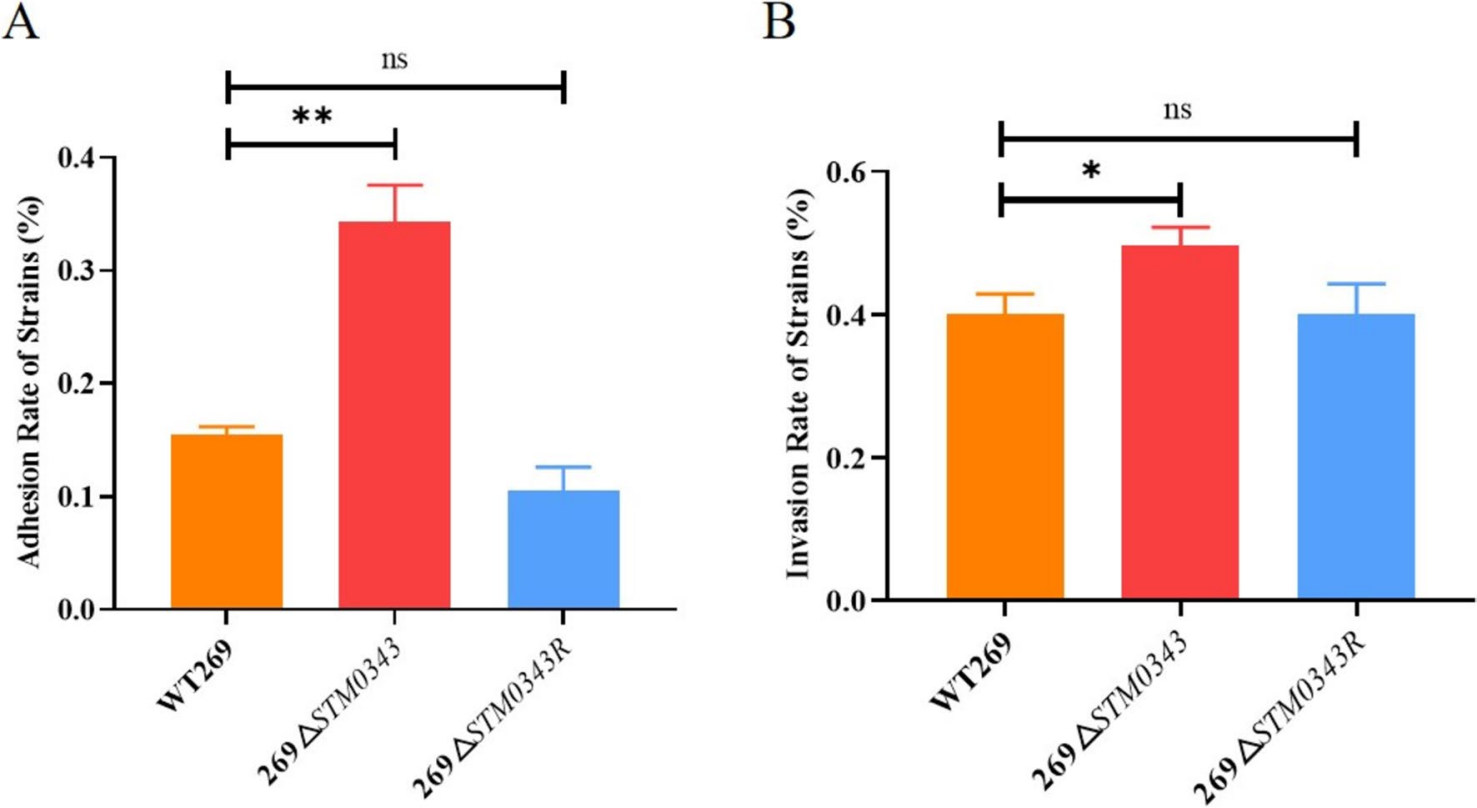
**Effect of *STM0343* on the cell invasion and adhesion abilities of *S.* Typhimurium.** **A** Characterisation of *STM0343* on the ability of the strain to adhesion to HeLa cells; **B** Characterisation of *STM0343* on the ability of the strain to invade HeLa cells. *P < 0.05, **P < 0.01.

This study evaluated the virulence of *S.* Typhimurium by conducting cell adhesion and invasion assays using HeLa cells as an in vitro experimental model. The results indicated that the mutant strain 269Δ*STM0343* exhibited a onefold increase in adhesion ability and a 25.67% increase in invasion ability compared to the wild-type strain WT269 (*P* < 0.05). Furthermore, the complemented strain 269Δ*STM0343R* restored both adhesion and invasion abilities to levels comparable to the wild strain (Figure 5). These findings tentatively suggest that *STM0343* may play a role in inhibiting the virulence of *S.* Typhimurium.

### Deletion of *STM0343* enhances the virulence of *S.* Typhimurium in a mouse model of infection

**Figure 6:**
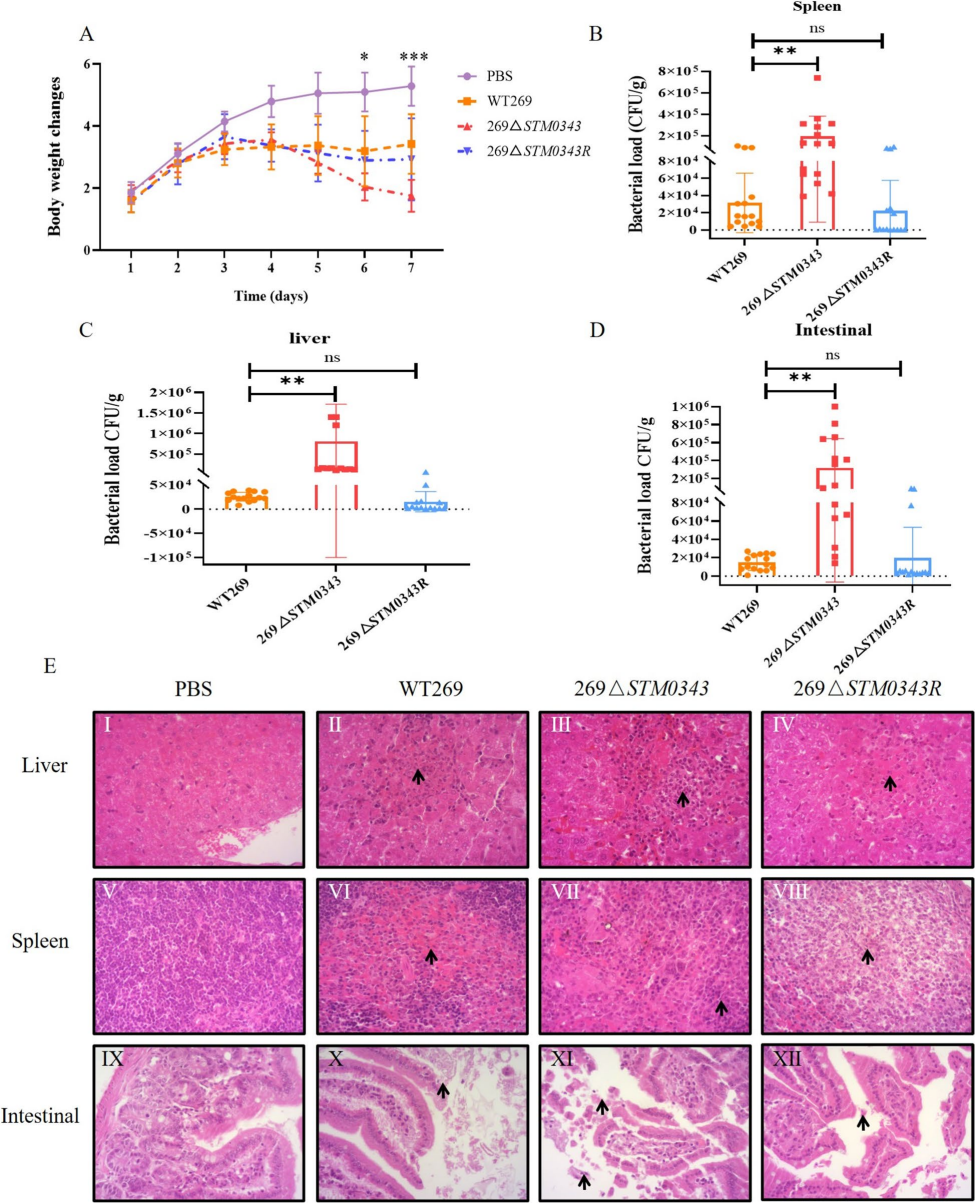
**Infection of mice with *S.* Typhimurium strains in vivo.** **A** Changes in body weight of mice stimulated by WT269, 269Δ*STM0343* and 269Δ*STM0343R*; **B**–**D** Bacterial load in the spleen, liver and intestine of experimental mice challenged by WT269, 269Δ*STM0343* and 269Δ*STM0343R*; **E** Histopathological section observations of livers, spleens and intestines of mice infected with WT269, 269Δ*STM0343* and 269Δ*STM0343R*, the black arrows represent lesions. *P < 0.05, **P < 0.01, ***P < 0.001.

To further investigate the role of *STM0343* in the pathogenesis of *S.* Typhimurium, this study conducted in vivo infection experiments using mice. First, the body weight of the mice was monitored for seven consecutive days following the challenge. The results indicated that the body weight of mice in the blank control group (PBS) exhibited a steady increase. In contrast, the body weight of mice in the WT269, 269Δ*STM0343,* and 269Δ*STM0343*R challenged groups was lower than that of the blank control group. The body weight of mice challenged with WT269 and 269Δ*STM0343R* increased initially over the first three days and then gradually stabilised. In contrast, the body weight of mice challenged with 269Δ*STM0343* began to decrease on day four and was significantly lower than that of the other two groups from day six onward (*P* < 0.05, Figure 6A).

The study also investigated the bacterial load in the spleen, liver, and intestine of the mice across the different groups. Compared to the WT269 challenge mice, the bacterial load in the intestine, spleen, and liver of mice infected with the 269Δ*STM0343* strain increased 21-fold, fivefold, and 30-fold, respectively (*P* < 0.01). However, there was no significant difference in bacterial load between the mice infected with the 269Δ*STM0343*R strain and those infected with the wild-type strain (*P* > 0.05, Figures 6B–D).

Additionally, the pathological examination of the internal organs revealed that the mice in the challenge groups experienced varying degrees of pathological changes in the liver, spleen, and intestine compared to the blank control group. Notably, the internal organs of mice challenged with the 269Δ*STM0343* strain showed more severe damage than those challenged with the WT269 and 269Δ*STM0343R* strains (Additional file 6).

The livers of the mice challenged with 269Δ*STM0343* displayed coagulation necrosis similar to that of the mice challenged with WT269, but they also exhibited significant inflammatory foci. In addition to the amyloid deposition observed in the spleen of the WT269 challenge mice, those challenged with 269Δ*STM0343* showed sparse lymphocytes and exposed endothelial cells in their spleens.

Examination of intestinal tissues revealed that all mice in the three challenge groups demonstrated necrosis and sloughing of intestinal epithelial cells. The intestinal damage in mice infected with the 269Δ*STM0343* strain was more severe compared to those challenged with the WT269 and 269Δ*STM0343*R strains. Mice exposed to the 269Δ*STM0343* strain exhibited denatured, necrotic, and detached intestinal mucosal epithelial cells, fragmented tissue, and loss of histological morphology (Figure 6E). These findings suggest that *STM0343* has a negative regulatory effect on the virulence of *S.* Typhimurium.

### *STM0343 *affects the virulence of *S.* Typhimurium by regulating the expression of *CsgB*

**Figure 7:**
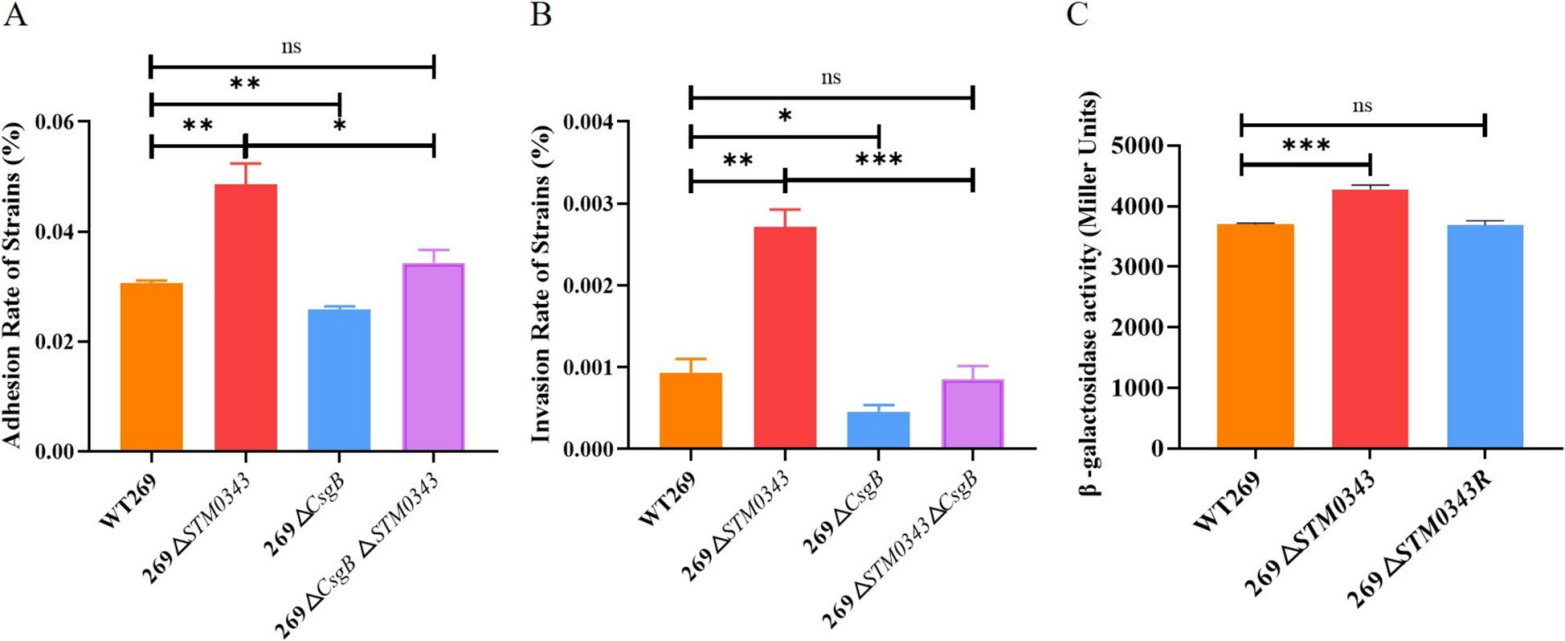
***STM0343* affects* S.* Typhimurium virulence by regulating *CsgB*.** **A**, **B** Cell adhesion and invasion assay. **C** LacZ reporter system to detect regulation of *CsgB* expression by *STM0343*. *P < 0.05, **P < 0.01, ***P < 0.001.

Based on the above findings, *STM0343* influences the expression of *CsgB*, which encodes curli fimbriae, a key virulence factor. To explore the relationship between *STM0343* and *CsgB* in regulating the virulence of *S.* Typhimurium, this study conducted adhesion and invasion assays on HeLa cells. The 269Δ*STM0343* mutant strain displayed significantly improved cell invasion and adhesion capabilities compared to the wild-type strain WT269. However, when *CsgB* was simultaneously deleted in the 269Δ*STM0343* background (i.e., 269Δ*STM0343*Δ*CsgB*, Additional file 7), both adhesion and invasion abilities decreased by 29.41% and 68.58%, respectively, compared to the 269Δ*STM0343* strain. These abilities returned to levels comparable to the wild-type strain (*P* > 0.05, Figures 7A and B), suggesting that *STM0343* regulates the virulence of *S.* Typhimurium through *CsgB*.

To further investigate whether *STM0343* has a regulatory effect on *CsgB* expression, we performed a LacZ transcriptional reporter assay (Additional file 8). We measured β-galactosidase activity as an indicator of *CsgB* activation. As expected, β-galactosidase activity was elevated by 15.59% in the 269Δ*STM0343* strain compared to the wild-type strain WT269 (*P* < 0.001). Additionally, in the complemented strain 269Δ*STM0343R*, β-galactosidase activity was restored to the level observed in WT269 (Figure 7C). These results suggest that *STM0343* inhibits the expression of *CsgB*, thereby reducing the virulence of *S.* Typhimurium.

## Discussion

Stress adaptation and effective virulence are key factors in the widespread occurrence of *S.* Typhimurium, presenting significant challenges for its prevention and control. A crucial regulatory molecule involved in these processes is c-di-GMP, which is commonly found in bacteria and regulates various life processes, including biofilm formation and virulence. Therefore, studying the genes involved in the c-di-GMP pathway is essential for identifying potential targets for preventing and controlling *S.* Typhimurium.

Preliminary transcriptomics studies, which involved constructing strains with varying abilities to form biofilms, revealed that c-di-GMP is essential for the biofilm formation of *S.* Typhimurium. Additionally, the gene *STM0343*, a putative EAL domain protein, was identified as a key player in the c-di-GMP pathway [17].

In this study, we further investigated the function of *STM0343*. Our results interesting showed that *STM0343* decreased c-di-GMP levels in bacteria, independent of bacterial growth. Previous studies have recognised *STM0343* as a potential EAL domain gene [26], corroborating earlier findings that EAL proteins can reduce c-di-GMP levels [11]. This suggests that *STM0343* may function as a phosphodiesterase. However, further experiments, such as point mutation analysis, are necessary to confirm this.

We further investigated the role that *STM0343* plays in the biofilm formation ability of *S.* Typhimurium. The crystal violet assay demonstrated that the deletion of *STM0343* enhanced biofilm formation. Additionally, the Congo red plate assay showed that the colony morphology of 269Δ*STM0343* appeared drier and rougher. This suggests an increased production of the extracellular matrix, which facilitates biofilm formation [27].

These results indicate that *STM0343* functions as an inhibitor of biofilm formation by *S.* Typhimurium. Biofilm is primarily composed of bacterial communities that are encased in an extracellular matrix, which includes exopolysaccharides, extracellular proteins and extracellular DNA (eDNA) [28]. To better understand *STM0343*’s regulatory role in *S.* Typhimurium biofilm formation, we quantified the extracellular matrix associated with the biofilm. We found that *STM0343* primarily inhibits the production of extracellular proteins and exopolysaccharides.

Extracellular proteins are crucial for stabilising the biofilm structure and for adhering bacteria to surfaces [28]. As a result, the levels of these extracellular proteins were elevated in the strain 269Δ*STM0343*. Furthermore, the deletion of *STM0343* caused a significant increase in the expression levels of the *CsgA* and *CsgB* genes, both of which are essential for encoding extracellular proteins [29, 30].

Exopolysaccharides are believed to play a crucial role in the adhesion of bacteria to biotic or abiotic surfaces during the formation of biofilms. In this study, we found that the exopolysaccharide content was significantly higher in the strain 269Δ*STM0343* compared to the wild type. Furthermore, cellulose, which is the main exopolysaccharide component of the biofilm matrix, along with its encoding genes *BcsA* and *BcsB*, showed increased levels of expression in the 269Δ*STM0343* strain.

The results indicated that *STM0343* reduces the ability of *S.* Typhimurium to form biofilms primarily by limiting the synthesis of extracellular proteins and exopolysaccharides. Interestingly, previous studies have shown that deleting several EAL domain genes does not impact biofilm formation [31]. This emphasises the critical role of *STM0343*, identified in this study, in regulating c-di-GMP levels and biofilm formation. Since biofilms are an important adaptive mechanism for bacteria [32], our findings underscore the necessity for further investigation into the mechanisms behind *STM0343*’s regulatory effects.

The deletion of *STM0343* was found to inhibit motility of *S.* Typhimurium, primarily due to a reduction in flagellar synthesis. This finding is supported by a significant decrease in *flhD* expression, a crucial regulator of flagellar synthesis, as noted in previous studies [33].

Interestingly, compensatory mechanisms may arise in response to the loss of *STM0343*. We observed an increase in the expression of fimbria-like adhesins (*CsgA* and *CsgB*). This finding is consistent with other research suggesting that when flagellar motility is compromised, bacteria often enhance their adhesive properties to maintain their ecological niches [34].

This shift highlights a strategic transition: motility allows bacteria to access optimal habitats, such as nutrient-rich environments [35], and facilitates their transition from a planktonic state to a stationary state, ultimately leading to biofilm formation [36]. Therefore, *STM0343* is a novel molecule that inhibits the biofilm formation of *S.* Typhimurium.

Biofilm formation provides a protective effect for *S.* Typhimurium; however, our findings indicate that *STM0343* inhibits biofilm formation. To further investigate the role of *STM0343* in the resistance of *S.* Typhimurium to adverse environmental factors, we first examined its effect on antibiotic resistance. The results showed that the strain 269Δ*STM0343* exhibited a significantly increased resistance to multiple antibiotics, including ceftazidime, ceftiofur, cefepime, cefotaxime, tetracycline, doxycycline, kanamycin, streptomycin, and nalidixic acid. Cephalosporins and fluoroquinolones are commonly used to treat severe *Salmonella* infections [37].

Previous studies have shown that bacteria capable of forming biofilms often have increased tolerance to antibiotics, making treatment more challenging [32]. Therefore, inhibiting biofilm formation by *STM0343* could be a critical factor in managing antibiotic resistance in *S.* Typhimurium.

In addition to antibiotic resistance, we found that the deletion of *STM0343* increased resistance to SDS disinfectants and enhanced tolerance to oxidative and acidic stress. SDS disinfectants are commonly used to eliminate environmental bacteria, while oxidative and acid stress act as antibacterial mechanisms in mammalian cells, helping to rapidly eliminate bacterial pathogens [38, 39]. Therefore, *STM0343* plays a crucial role in eliminating stress adaptation in *S.* Typhimurium.

Our study revealed that *STM0343* plays a role in regulating the virulence of *S.* Typhimurium. Firstly, through in vitro cell invasion and adhesion assays, we found that the deletion of *STM0343* significantly enhanced adherence to HeLa cells compared to the wild-type strain. This increased adherence ability allows the bacteria to persist in the extracellular space and facilitates their invasion into host cells [40]. Interestingly, the deletion of *STM0343* enhanced the invasion ability of *S.* Typhimurium to HeLa cells, indicating that *STM0343* may inhibit the virulence of *S.* Typhimurium.

Additionally, our in vivo experiments using a mouse model support this conclusion. Mice treated with 269Δ*STM0343* exhibited noticeable weight loss, and there was a significant increase in bacterial load in the intestines, liver, and spleen, along with greater tissue damage in these organs compared to the wild-type strain WT269. Effective virulence is essential for *S.* Typhimurium to disseminate widely, and the coordinated expression of genes in pathogenic bacteria is necessary to enhance the efficiency of virulence processes [19].

Our findings emphasise the significant role of *STM0343* in regulating the virulence of *S.* Typhimurium.

Our study investigated the mechanism by which *STM0343* regulates virulence. We found that *STM0343* influences the expression of *CsgB*, a regulatory gene crucial for the synthesis of curli fimbriae and an important factor in virulence [41]. Previous studies have identified key factors that affect the pathogenicity of *S.* Typhimurium by regulating downstream virulence genes [42]. Based on this information, we hypothesised that *STM0343* modulates the virulence of *S.* Typhimurium by regulating *CsgB*.

To test this hypothesis, we created a double deletion mutant of *STM0343* and *CsgB* (269Δ*STM0343*Δ*CsgB*). Our observations indicated that the enhanced pathogenicity of 269Δ*STM0343* was significantly reduced and returned to the levels seen in the wild-type strain after the simultaneous deletion of *CsgB* (269Δ*STM0343*Δ*CsgB*).

To further confirm the regulatory effect of *STM0343* on *CsgB*, we conducted a LacZ reporter gene fusion assay to evaluate *CsgB* activity by indirectly measuring β-galactosidase levels [43]. The results showed that the β-galactosidase content in the *STM0343* knockout mutant was significantly higher than that in the wild-type WT269. Additionally, reintroducing the *STM0343* gene restored β-galactosidase levels to those of the wild-type. This finding suggests that *STM0343* can repress the expression of the *CsgB*.

These findings are consistent with the qRT-PCR results mentioned above and further support the conclusion that *STM0343* reduces the pathogenicity of *S.* Typhimurium by inhibiting the expression of the curli fimbriae gene *CsgB*. In summary, this study has identified the key gene *STM0343* that regulates the stress adaptability and virulence of *S.* Typhimurium. Future research should explore the molecular mechanisms that underlie the regulatory factors influencing the optimal biological functions of *STM0343*. This understanding will be valuable in addressing the challenges of controlling *S.* Typhimurium from the perspective of stress adaptability.

Stress adaptation and virulence are critical for the survival and proliferation of *S.* Typhimurium. In this study, we identified a novel EAL domain gene, *STM0343*, which reduces the levels of c-di-GMP while also affecting the stress resistance and virulence of *S.* Typhimurium. Regarding stress resistance, *STM0343* enhances bacterial motility by promoting the expression of flagellar synthesis genes. Additionally, it suppresses the production of extracellular proteins by downregulating the expression of *CsgB* and *CsgA*, as well as exopolysaccharides, by decreasing the expression of *BcsA* and *BcsB*. This, in turn, inhibits biofilm formation. Ultimately, *STM0343* decreases the resistance of *S.* Typhimurium to various antibiotics, acids, oxidative stress, and disinfectants. Additionally, *STM0343* diminishes the virulence of *S.* Typhimurium by inhibiting the expression of the virulence factor *CsgB*. This study represents a significant advancement in the functional identification of c-di-GMP pathway genes and opens up new possibilities for developing prevention and control strategies against *S.* Typhimurium.

## Supplementary Information

**Additional file 1: List of primers used for PCR.**

**Additional file 2: List of primers used for qRT-PCR.**

**Additional file 3: List of plasmids used in this work.**

**Additional file 4: Construction of *STM0343* deletion mutant and deletion complementation strains.** **A** Identification of the *STM0343* deletion mutation, lane 1 is the successful mutant strain with a band size of 740bp, lane 2 is the wild strain with a band size of 2320bp. **B** and **C** Construction of the *STM0343* complementation strain, **B** Construction of *STM0343* expression vector using plasmid pBAD as a vector, lane 1 is an empty vector, and the band size is 420bp, lane 2 for the successful construction of the expression vector, with a band size of 2100 bp. **C** Validation of successful transfer of *STM0343* expression vector into the strain. Lanes 1, 2, and 3 are all successfully constructed *STM0343* complementation strains with a band size of 2100bp. M: DL5000 DNA Marker.

**Additional file 5: qRT-PCR analysis of the effect of *STM0343* on gene expression related to biofilm formation and motility.**

**Additional file 6: Pathology scores in liver, spleen, and intestinal tissues of WT269, 269Δ*STM0343*, and 269Δ*STM0343R*-infected mice.** *P < 0. 05, ***P < 0.001.

**Additional file 7: Construction of *CsgB* deletion strain, *STM0343* and CsgB double deletion strain.** **A** Identification of *CsgB* deletion strains, lanes 1–10 are successful deletion of *CsgB* strains. **B** Deletion of *CsgB* in the genetic background of 269Δ*STM0343*, lanes 1–10 are successfully constructed *CsgB* and *STM0343* double deletion mutants (269Δ*CsgB*Δ*STM0343*). + represents WT269 as a positive control, M: DL1000 DNA Marker.

**Additional file 8: Construction of the LacZ gene reporter system.** **A** PCR amplification of the *CsgB* promoter. 1, 2, 3 are successfully amplified samples with band sizes of 885bp, M: DL1000 DNA Marker; **B** Purification of *CsgB* promoter after double enzyme digestion, M: DL5000 DNA Marker; **C** Plasmid PRCL purification after double digestion, M: DL5000 DNA Marker; **D** PCR validation of the recombinant plasmid PRCL-*CsgB* after transformation into WT269, 269Δ*STM0343*, 269Δ*STM0343*R, 1–4 represent the successful transfer of recombinant plasmid into the WT269; 5–8 represent the successful transfer of recombinant plasmid into the 269Δ*STM0343*; numbers 9–14 represent the successful transfer of recombinant plasmid into the 269Δ*STM0343*R. + , for plasmid PRCL amplification band, M: DL2000 DNA Marker.

## Data Availability

All data generated or analysed during this study are included in this published article and its supplementary information files.
