# Supplementary material for: Newly identified c-di-GMP pathway putative EAL domain gene STM0343 regulates stress resistance and virulence in Salmonella enterica serovar Typhimurium
Source: Vet Res. 2025 Jan 15;56:13. doi: 10.1186/s13567-024-01437-0 (PMC11737180; doi:10.1186/s13567-024-01437-0)
Supplement: Supplementary file 1 — Additional file 1: List of primers used for PCR. [file 13567_2024_1437_MOESM1_ESM.docx]

**Additional file 1. List of primers used for PCR**

| Primer Name | Sequence 5’-3’ | Purpose |
| --- | --- | --- |
| *STM0343*-L1-F | GTTATCCTATCAAAGCGTACAGTGGCAAAAGGAAGGCCGGTAAGAGTGTAGGCTGGAGCTGCTTC | used to amplify kanamycin resistance genes containing FRT sites and upstream and downstream homologous fragments of *STM0343* |
| *STM0343*-L1-R | GTTATCCTATCAAAGCGTACAGTGGCAAAAGGAAGGCCGGTAAGAGTGTAGGCTGGAGCTGCTTC |  |
| *CsgB*-L2-F | TCGTTTTATTAGCACTTTGGTATGAGCTTAAATAACAAAATACCACGCGTGGGTGGTGTAGGCTGGAGCTGCTTC | used to amplify kanamycin resistance genes containing FRT sites and upstream and downstream homologous fragments of *CsgB* |
| *CsgB*-L2-R | TTTTAAAAGTTTCATGGTAAAACCCCCATCGGATTGATTTAAAAGTCGTAACGGTACATATGAATATCCTCCTTAG |  |
| *STM0343*-JD-F | TGATACTGCTGGCGATTT | To identify the successful deletion mutation of *STM0343* |
| *STM0343*-JD-R | AGACCACGACACCTTTGC |  |
| *CsgB*-JD-F | ATGGGAAGCATAAGAACA | To identify the successful deletion mutation of *CsgB* |
| *CsgB*-JD-R | CACTGCCAGAAACTACGA |  |
| pKD46-JD-F | GCAACTTTATCCGCCTCC | Identify the presence of plasmid pKD46 |
| pKD46-JD-R | TCGCCCTTATTCCCTTTT |  |
| pCP20-JD-F | CAGTGCTGCAATGATACCGC | Identify the presence of plasmid pCP20 |
| pCP20-JD-R | TCCTTGAGAGTTTTCGCCCC |  |
| *CsgB*-pro-F | CCCAAGCTTGGGCCGGCCGAGATATCTTCCAGAGAAC | For amplification of the *CsgB* promoter region |
| *CsgB*-pro-R | CGGGATCCCGAGCGAAAATTATCTATTACCTTGTT |  |
| PRCL-CMS-F | TGAGGGGACGACGACAGTAT | Used to characterise the successful construction of the LacZ reporting system |
| PRCL-CMS-R | TGTTGTTTGTCGGTGAACGC |  |
