# Supplementary material for: Newly identified c-di-GMP pathway putative EAL domain gene STM0343 regulates stress resistance and virulence in Salmonella enterica serovar Typhimurium
Source: Vet Res. 2025 Jan 15;56:13. doi: 10.1186/s13567-024-01437-0 (PMC11737180; doi:10.1186/s13567-024-01437-0)
Supplement: Supplementary file 2 — Additional file 2: List of primers used for qRT-PCR. [file 13567_2024_1437_MOESM2_ESM.docx]

**Additional file 2. List of primers used for qRT-PCR**

| Primer Name | Sequence 5’-3’ |
| --- | --- |
| 16S rRNA-QF | CCAGGGCTACACACGTGCTA |
| 16S rRNA-QR | TCTCGCGAGGTCGCTTCT |
| *BcsA*-QF | TGCGGGCTGATTCTGCTGTTTGC |
| *BcsA*-QR | CGAAATGATTCACGCCCGCCGT |
| *BcsB*-QF | AAAAGGTATCGCACAAGGG |
| *BcsB*-QR | GCTACGCAGCAAATAGAGGT |
| *CsgA*-QF | CCAGGGTGCGGATAACAGTA |
| *CsgA*-QR | CCAACCTGACGCACCATTAC |
| *CsgB*-QF | TCGACTTTCGCCCGATTAT |
| *CsgB*-QR | AGGTCCAGGGTGACAGCAT |
| *flhC*-QF | GAAAGTGGGTTGCTTGAATTG |
| *flhC*-QR | GCATCTCGGGAAAGTTTACG |
| *flhD*-QF | TGATGATCGTCAAACCGGAAA |
| *flhD*-QR | TGCCGCAGATGGTCAAACTG |
