# Supplementary material for: Newly identified c-di-GMP pathway putative EAL domain gene STM0343 regulates stress resistance and virulence in Salmonella enterica serovar Typhimurium
Source: Vet Res. 2025 Jan 15;56:13. doi: 10.1186/s13567-024-01437-0 (PMC11737180; doi:10.1186/s13567-024-01437-0)
Supplement: Supplementary file 3 — Additional file 3: List of plasmids used in this work. [file 13567_2024_1437_MOESM3_ESM.docx]

**Additional file 3. List of plasmids used in this work**

| Plasmids Name | Purpose |
| --- | --- |
| pKD46 | Used to promote homologous recombination. |
| pKD4 | Used to amplify homologous targeting fragments of target genes containing kanamycin resistance. |
| pCP20 | Eliminate the Kanamycin-resistant fragment of PKD4 left after homologous recombination with the target gene. |
| pBAD-HisA | An expression vector for the construction of complementation strains with gene deletion mutations. |
| pRCL | This plasmid was used for the LacZ reporter gene fusion experiments. |
