# Supplementary figures and images for: Newly identified c-di-GMP pathway putative EAL domain gene STM0343 regulates stress resistance and virulence in Salmonella enterica serovar Typhimurium
Source: Vet Res. 2025 Jan 15;56:13. doi: 10.1186/s13567-024-01437-0 (PMC11737180; doi:10.1186/s13567-024-01437-0)

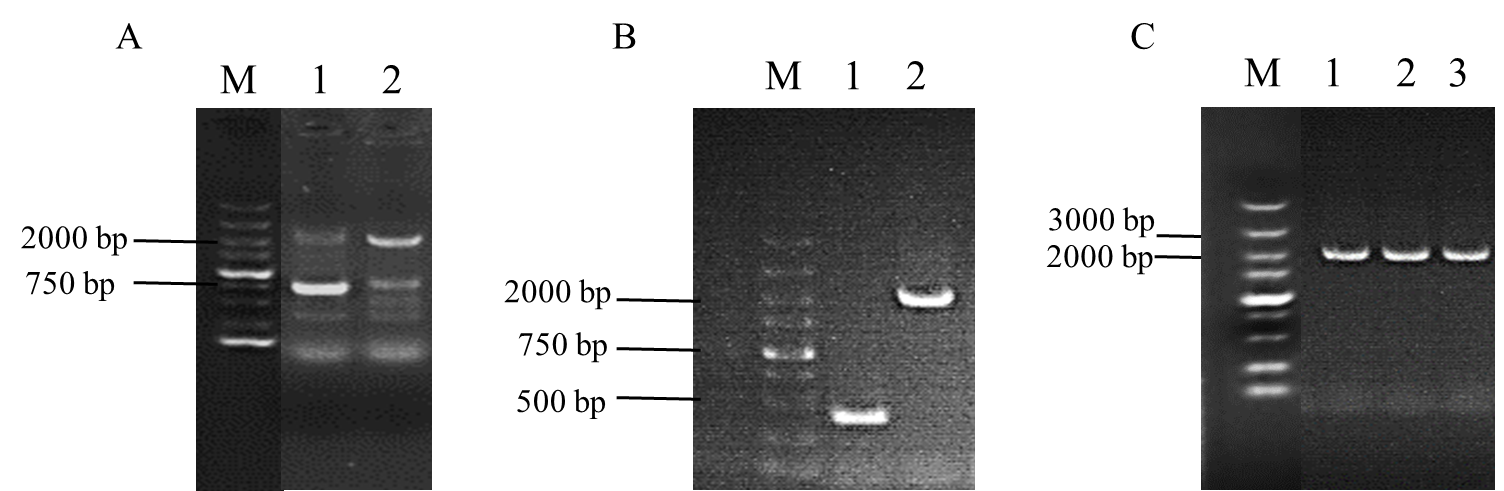

Supplement: Supplementary file 4 — Additional file 4: Construction of STM0343 deletion mutant and deletion complementation strains. A Identification of the STM0343 deletion mutation, lane 1 is the successful mutant strain with a band size of 740bp, lane 2 is the wild strain with a band size of 2320bp. B and C Construction of the STM0343 complementation strain, B Construction of STM0343 expression vector using plasmid pBAD as a vector, lane 1 is an empty vector, and the band size is 420bp, lane 2 for the successful construction of the expression vector, with a band size of 2100 bp. C Validation of successful transfer of STM0343 expression vector into the strain. Lanes 1, 2, and 3 are all successfully constructed STM0343 complementation strains with a band size of 2100bp. M: DL5000 DNA Marker. [file 13567_2024_1437_MOESM4_ESM.docx]

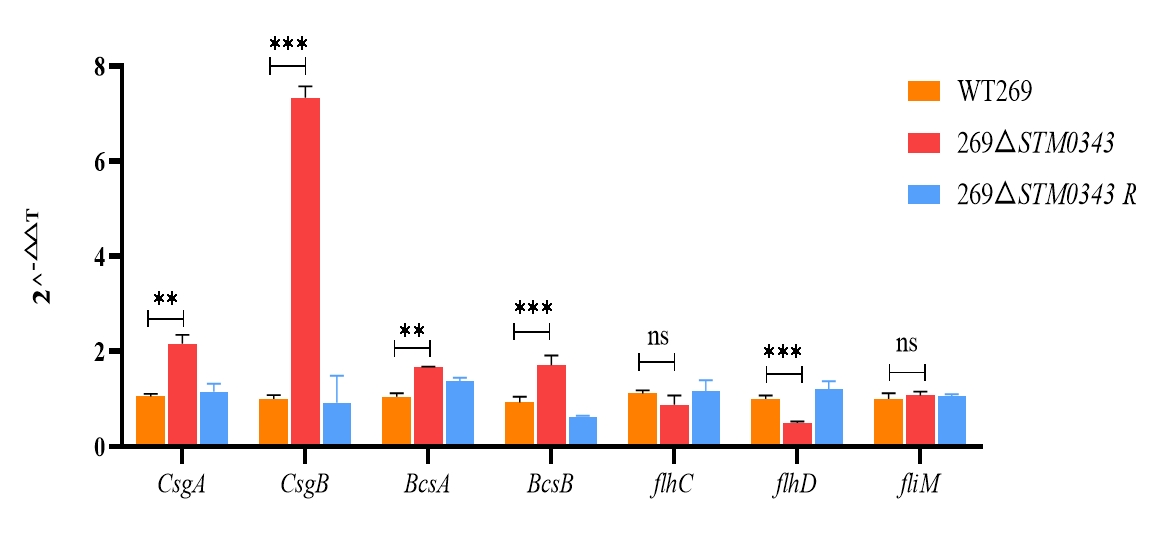

Supplement: Supplementary file 5 — Additional file 5: qRT-PCR analysis of the effect of STM0343 on gene expression related to biofilm formation and motility. [file 13567_2024_1437_MOESM5_ESM.docx]

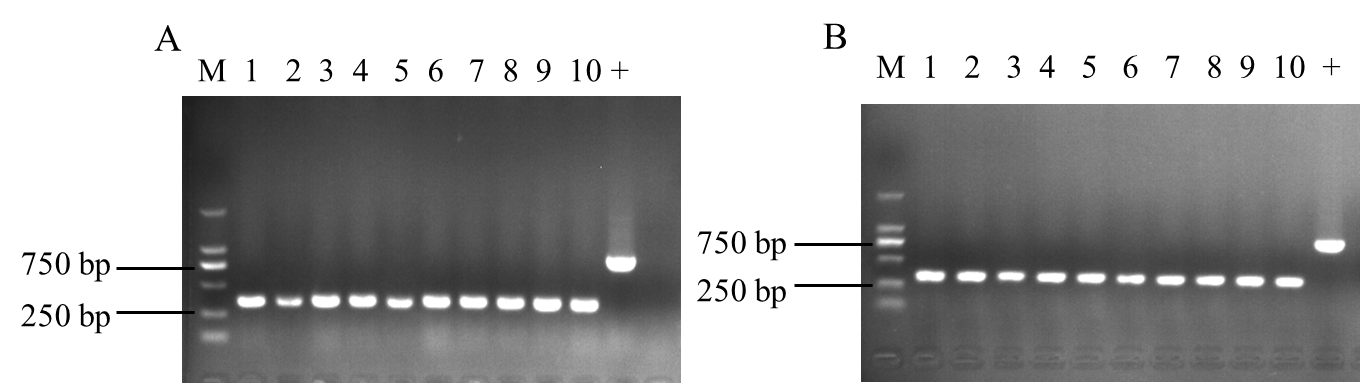

Supplement: Supplementary file 7 — Additional file 7: Construction of CsgB deletion strain, STM0343 and CsgB double deletion strain. A Identification of CsgB deletion strains, lanes 1–10 are successful deletion of CsgB strains. B Deletion of CsgB in the genetic background of 269ΔSTM0343, lanes 1–10 are successfully constructed CsgB and STM0343 double deletion mutants (269ΔCsgBΔSTM0343). + represents WT269 as a positive control, M: DL1000 DNA Marker. [file 13567_2024_1437_MOESM7_ESM.docx]

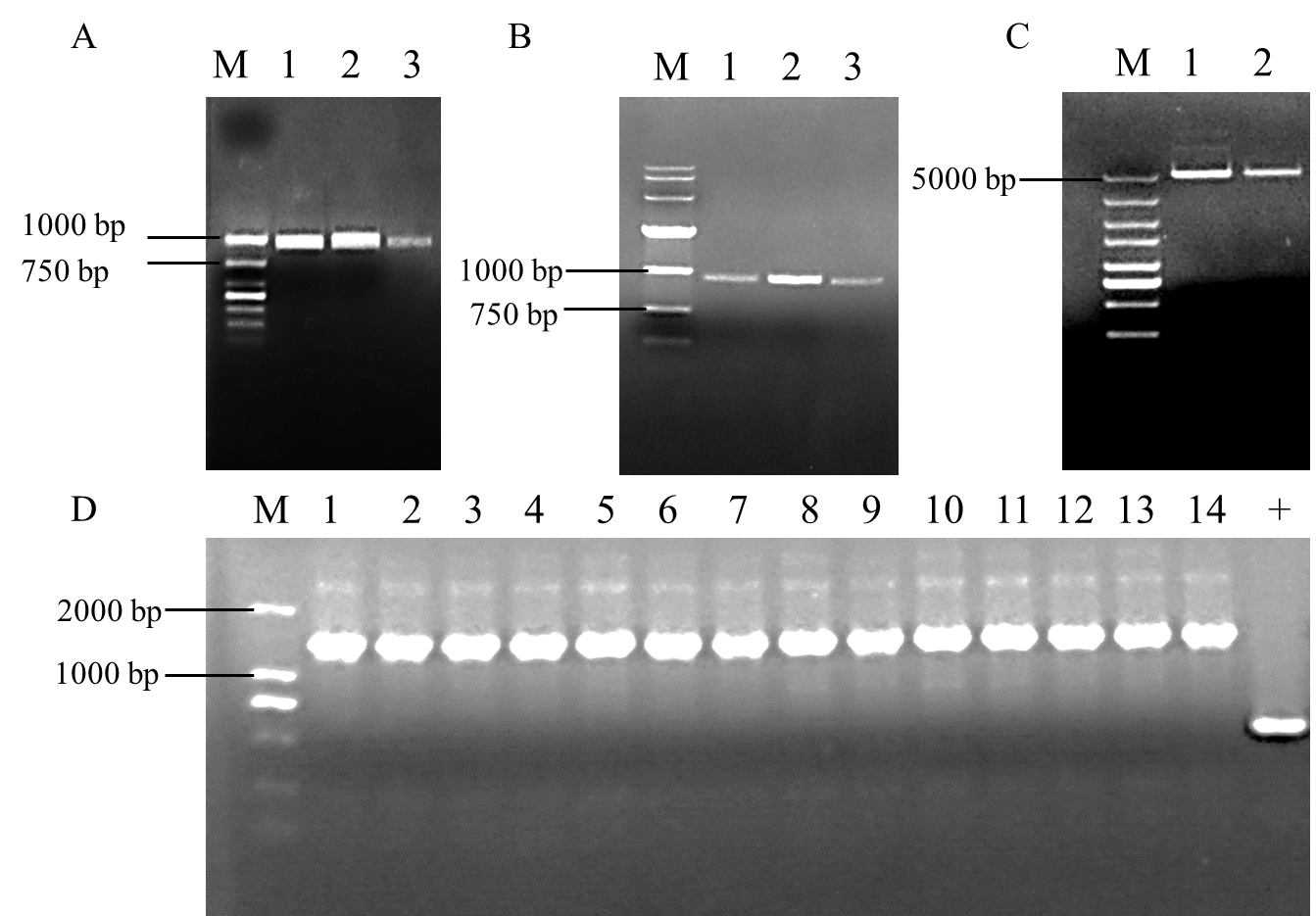

Supplement: Supplementary file 8 — Additional file 8: Construction of the LacZ gene reporter system. A PCR amplification of the CsgB promoter. 1, 2, 3 are successfully amplified samples with band sizes of 885bp, M: DL1000 DNA Marker; B Purification of CsgB promoter after double enzyme digestion, M: DL5000 DNA Marker; C Plasmid PRCL purification after double digestion, M: DL5000 DNA Marker; D PCR validation of the recombinant plasmid PRCL-CsgB after transformation into WT269, 269ΔSTM0343, 269ΔSTM0343R, 1–4 represent the successful transfer of recombinant plasmid into the WT269; 5–8 represent the successful transfer of recombinant plasmid into the 269ΔSTM0343; numbers 9–14 represent the successful transfer of recombinant plasmid into the 269ΔSTM0343R. + , for plasmid PRCL amplification band, M: DL2000 DNA Marker. [file 13567_2024_1437_MOESM8_ESM.docx]
