# Supplementary material for: Newly identified c-di-GMP pathway putative EAL domain gene STM0343 regulates stress resistance and virulence in Salmonella enterica serovar Typhimurium
Source: Vet Res. 2025 Jan 15;56:13. doi: 10.1186/s13567-024-01437-0 (PMC11737180; doi:10.1186/s13567-024-01437-0)
Supplement: Supplementary file 5 [file 13567_2024_1437_MOESM6_ESM.docx]

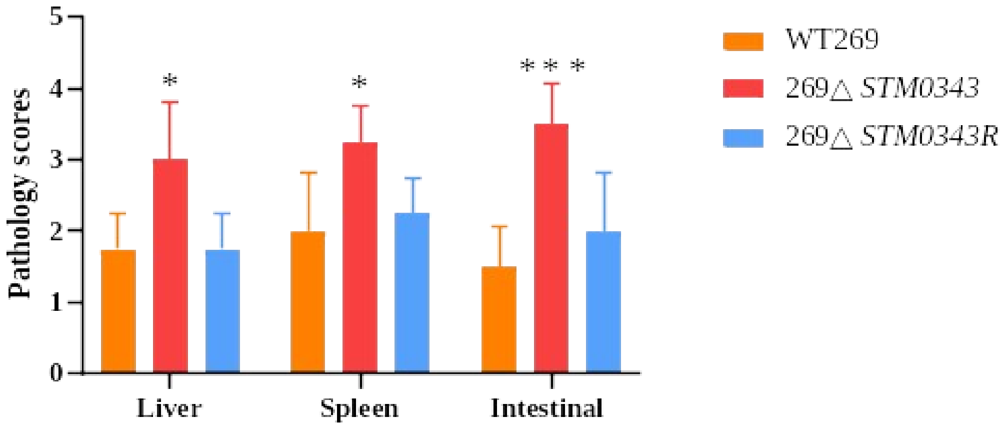

Pathology scores
0
1
2
3
4
5
*
*
* * *
Liver
Spleen
Intestinal
WT269
269△ STM0343
269△ STM0343R
